# Dysregulated miR-671-5p / CDR1-AS / CDR1 / VSNL1 axis is involved in glioblastoma multiforme

**DOI:** 10.18632/oncotarget.6621

**Published:** 2015-12-15

**Authors:** Davide Barbagallo, Angelo Condorelli, Marco Ragusa, Loredana Salito, Mariangela Sammito, Barbara Banelli, Rosario Caltabiano, Giuseppe Barbagallo, Agata Zappalà, Rosalia Battaglia, Matilde Cirnigliaro, Salvatore Lanzafame, Enrico Vasquez, Rosalba Parenti, Federico Cicirata, Cinzia Di Pietro, Massimo Romani, Michele Purrello

**Affiliations:** ^1^ Dipartimento di Scienze Biomediche e Biotecnologiche, Sezione di Biologia e Genetica *G Sichel*, Unità di BioMedicina Molecolare, Genomica e dei Sistemi Complessi, Università di Catania, Catania, Italy, EU; ^2^ UOS Epigenetica dei Tumori, IRCCS A.O.U. San Martino-IST, Genova, Italy, EU; ^3^ Dipartimento di Scienze Mediche, Chirurgiche e Tecnologie Avanzate *G.F. Ingrassia*, Università di Catania, Catania, Italy, EU; ^4^ Dipartimento di Scienze Biomediche e Biotecnologiche, Sezione di Fisiologia, Università di Catania, Catania, Italy, EU

**Keywords:** glioblastoma multiforme (GBM), non coding RNAs (ncRNAs), microRNAs (miRNAs), circular RNAs (circRNAs), cell networks

## Abstract

MiR-671-5p is encoded by a gene localized at 7q36.1, a region amplified in human glioblastoma multiforme (GBM), the most malignant brain cancer. To investigate whether expression of miR-671-5p were altered in GBM, we analyzed biopsies from a cohort of forty-five GBM patients and from five GBM cell lines. Our data show significant overexpression of miR-671-5p in both biopsies and cell lines. By exploiting specific miRNA mimics and inhibitors, we demonstrated that miR-671-5p overexpression significantly increases migration and to a less extent proliferation rates of GBM cells. Through a combined *in silico* and *in vitro* approach, we identified CDR1-AS, CDR1, VSNL1 as downstream miR-671-5p targets in GBM. Expression of these genes significantly decreased both in GBM biopsies and cell lines and negatively correlated with that of miR-671-5p. Based on our data, we propose that the axis miR-671-5p / CDR1-AS / CDR1 / VSNL1 is functionally altered in GBM cells and is involved in the modification of their biopathological profile.

## INTRODUCTION

According to the World Health Organization (WHO), gliomas are grouped into 4 histological grades (Grade I to IV) that are defined by increasing degree of anaplasia, undifferentiation, aggressiveness [1, 2]. GBM is the most prevalent and aggressive cancer originating in the central nervous system, mainly in the brain [3]. Its prognosis is very poor, as GBM patients have a median overall survival of only 14 months. Overall age-adjusted incidence rates for all gliomas (ICD-O-3 morphology codes 9380–9480), normalized to the national population of each respective study, range from 4.67 to 5.73 / 100000 persons. Age-adjusted incidence of GBM (ICD-O-3 morphology codes 9440–9442, WHO grade IV) ranges from 0.59 to 3.69 / 100000 persons [3]. Data on the most common somatic mutations and corresponding pathways have demonstrated a very high molecular heterogeneity of GBM tumors [4–6]. MicroRNAs (miRNAs) are known to play an important role in GBM pathogenesis and progression [7–9]. A recently discovered class of non-coding RNAs, known as circular RNAs (circRNAs), has been established as a new post-transcriptional gene expression regulatory layer [10, 11]. CircRNAs are covalently closed non-coding RNAs, which regulate gene expression by acting as miRNA sponges, reducing their availability within the cell [11]. Cerebellar Degeneration-Related Protein 1 (CDR1) antisense (CDR1-AS, a miR-7 sponge also known as ciRS-7) is the only circRNA known to be targeted and degraded by a microRNA (miR-671-5p) [11]. Similar to its sense counterpart CDR1, it is highly expressed in the brain. It positively controls the expression of CDR1 mRNA, probably by stabilizing it through direct interaction [12]. It also functions as miR-7 sponge by sequestering it through multiple binding sites [11, 12]. This molecular system is regulated by miR-671-5p, which under appropriate conditions leads to Argonaute RISC catalytic component 2 (AGO2) - mediated degradation of CDR1-AS [12]. The human gene encoding miR-671 maps at 7q36.1, a genomic region frequently amplified in GBM [13]. We sought to verify the involvement of miR-671 in GBM pathogenesis by applying the gene candidate approach. By exploiting specific miRNA mimics and inhibitors, we demonstrate here that its overexpression in GBM biopsies and cell lines contributes to increase migration and proliferation rates of GBM cells. Based on our data, we propose that the axis miR-671-5p / CDR1-AS / CDR1 / Visinin-like 1 (VSNL1) is functionally altered in GBM and suggest that miR-671-5p is a novel oncomiR in GBM.

## RESULTS

### MiR-671-5p and miR-671-3p expression in GBM biopsies

**Figure 1:**
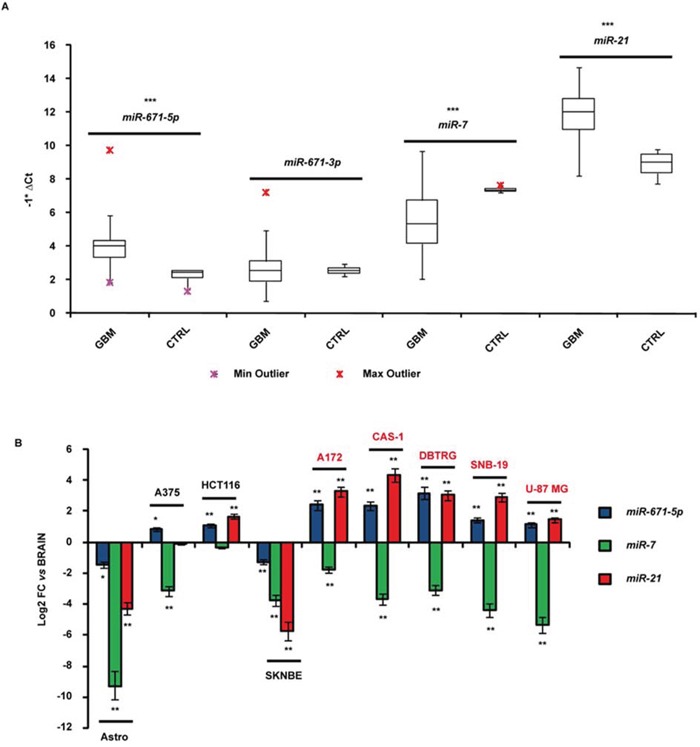
Expression of miR-671-5p, miR-671-3p, miR-21 and miR-7 in GBM biopsies **A.** and cell lines **B.** Expression values are reported as box plots with whiskers from minimum to maximum to represent −1*ΔCt, both in GBM biopsies as in controls (A), and as mean of fold change (FC) ± Standard Deviation versus normal brain (B). miR-99a and miR-92a were used as reference genes in experiments on biopsies and cell lines, respectively. *p-value < 0.05, **p-value < 0.01, ***p-value < 0.001, Student's *t*-test (*n* = 3).

MiR-671-5p resulted significantly overexpressed in GBM biopsies compared to all reference tissues (average fold change = 13-fold, *p*-value < 0.001, Student's *t*-test). MiR-671-3p expression did not significantly differ compared to controls. We also observed a significant overexpression of miR-21 (average fold change = 13.3-fold, *p*-value < 0.001, Student's *t*-test) and a significant underexpression of miR-7 (average fold change = −7.2-fold, *p*-value < 0.001, Student's *t*-test) in the same biopsies (Figure 1A).

### MiR-671-5p expression in GBM cell lines

Mir-671-5p resulted more than twofold overexpressed in A172, CAS-1, DBTRG, SNB-19 and U-87 MG GBM cells compared to whole brain, astrocytes and the neuroblastoma cell line SK-N-BE (Figure 1B). Three out of the five GBM cell lines (A172, CAS-1, DBTRG) showed more than twofold miR-671-5p overexpression also respect to other two tumor cell lines (A375, HCT116) (Figure 1B). All GBM cell lines showed under - and overexpression of miR-7 and miR-21 respectively, compared to whole brain, as reported by literature (Figure 1B).

### CDR1-AS, CDR1, CHPF2, VSNL1 expression in GBM biopsies

**Figure 2:**
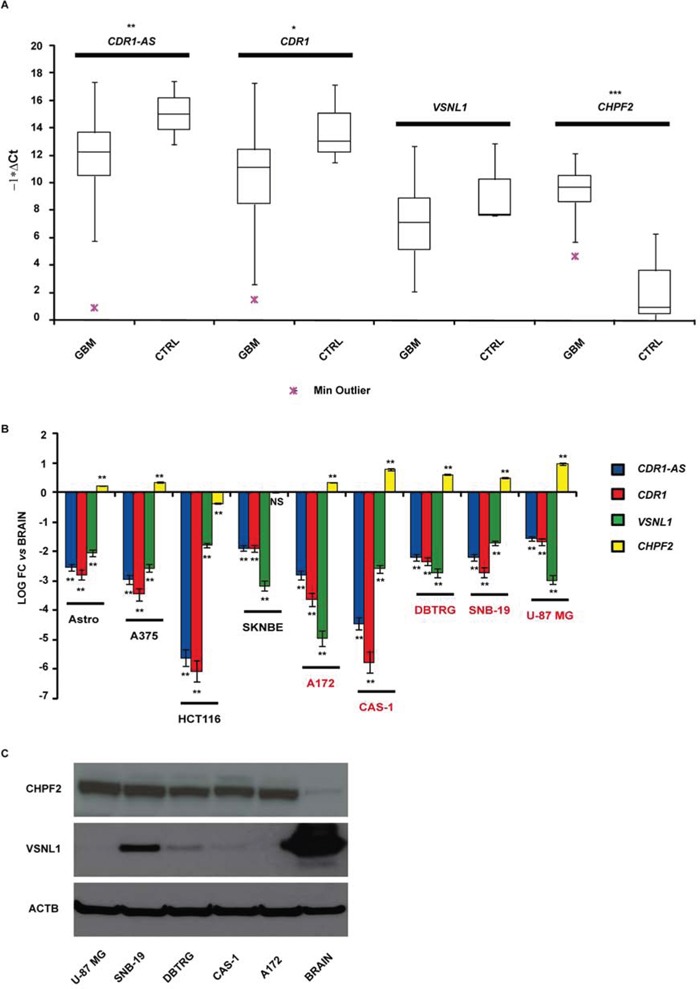
CDR1-AS, CDR1, VSNL1 and CHPF2 expression in GBM biopsies **A.** and cell lines **B.** Expression values are reported as box plots with whiskers from minimum to maximum to represent −1*ΔCt, both in GBM biopsies and controls (A), and as mean of fold change (FC) ± Standard Deviation versus normal brain (B). Western blot of CHPF2 and VSNL1 in GBM cell lines and normal brain tissue **C.** *p-value < 0.05, **p-value < 0.01, ***p-value < 0.001, Student's *t*-test (*n* = 3).

We identified 46 validated and 61 predicted targets of miR-671-5p (see Supplementary Tables 1 and 2): among them, we selected CDR1-AS, CHPF2 and VSNL1 for further analysis. CDR1-AS is a validated miR-671-5p target with intriguing gene expression regulatory features (see Introduction on circRNAs). CHPF2 is the host gene of miR-671-5p and there is some experimental evidence that is targeted by the same miRNA. Among the top 15 predicted targets (ordered by increasing mirSVR score), VSNL1 is a known tumor-suppressor gene regulating cell migration in several cancer types. We added CDR1 as further putative miR-671-5p target because its expression is known to be positively regulated by CDR1-AS (see Introduction and Discussion). Expression of the selected putative targets was analyzed in GBM biopsies and compared to normal brain parenchyma. We observed: (1) downregulation of CDR1 (average fold change = −2.84-fold; *p*-value = 0.028, Student's *t*-test) and CDR1-AS (average fold change = −3.51-fold, *p*-value = 0.008, Student's *t*-test); (2) upregulation of CHPF2 (average fold change > 100-fold, *p*-value < 0.001, Student's *t*-test); downregulation of VSNL1 (average fold change = −2.1-fold, *p*-value = 0.107, Student's *t*-test), statistically not significant (Figure 2A). CDR1 appeared significantly less expressed in males compared to females within our cohort (*p* = 0.027, Student's *t*-test). A negative correlation (statistically not significant) was observed between tumor size and CDR1 expression (*r* = −0.24, *p* = 0.094, Spearman Rank-Order Correlation test). We did not observe any other correlation between the expression of miR-671-5p or its targets and the clinical features of our GBM cohort.

### CDR1-AS, CDR1, CHPF2, VSNL1 expression in GBM cell lines

CDR1-AS and CDR1 resulted on average downregulated in GBM cell lines with respect to astrocytes and other cancer cell lines, with the only exception of HCT 116; CAS-1 showed the most impressive downregulation of CDR1-AS and CDR1. VSNL1 downregulation was common to all GBM cell lines and, on average, more pronounced with respect to other cancer cell lines, with the only exception of SN-K-BE. CHPF2 was overexpressed more than twofold in all GBM cell lines: similar to miR-671-5p, its overexpression appeared more pronounced in GBM cell lines than in other tissues (Figure 2B). Data on VSNL1 underexpression and CHPF2 overexpression in GBM cell lines were confirmed also at protein level, by using normal cerebral cortex as control tissue (Figure 2C).

### Negative correlation between expression of miR-671-5p and of CDR1-AS, CDR1 and VSNL1 in GBM biopsies and cell lines

**Figure 3:**
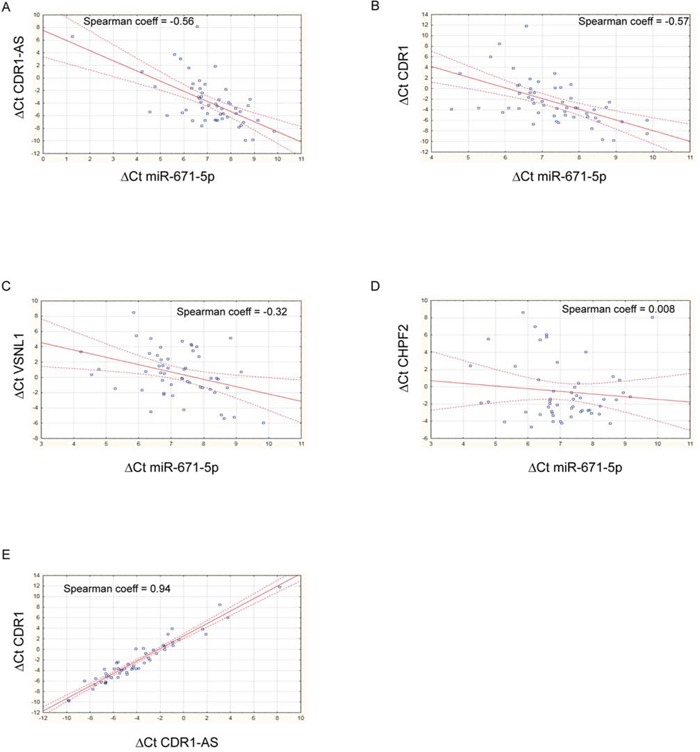
Scatter plots showing correlation between expression of miR-671-5p and its targets Spearman's nonparametric rank correlation coefficients were calculated using ΔCt values of miR-671-5p and its targets CDR1-AS **A.** CDR1 **B.** VSNL1 **C.** CHPF2 **D.** and ΔCt values of CDR1-AS and CDR1 **E.** See text for details.

**Figure 4:**
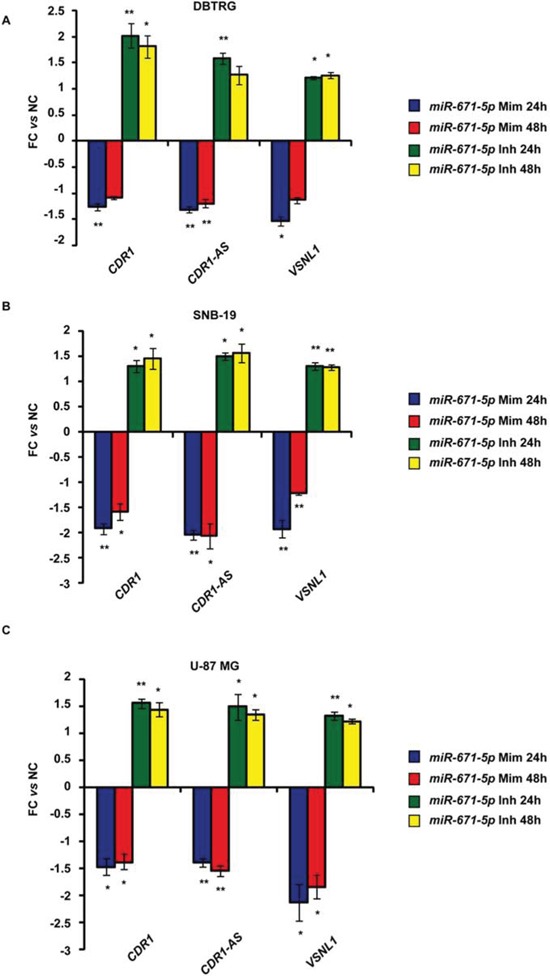
Expression of CDR1-AS, CDR1, VSNL1 in DBTRG, SNB-19, U-87 MG cell lines after transfection with miR-671-5p mimics (miR-671-5p Mim) or inhibitors (miR-671-5p Inh) Expression values are reported as mean of fold change (FC) ± Standard Deviation versus scramble molecules (NC) - transfected cells. *p-value < 0.05; **p-value < 0.01, Student's *t*-test (*n* = 3).

Expression of miR-671-5p negatively correlated with that of CDR1-AS, CDR1, VSNL1 (*r* = −0.56, −0.57, −0.32, *p* = 1.33e-05, 1.91e-05, 0.021, respectively; *n* = 54, 51, 52, respectively, Spearman's Rank-Order Correlation test) (Figure 3). A highly positive correlation was detected between CDR1-AS and CDR1 expression (*r* = 0.938, *p* = 0, *n* = 51, Spearman's Rank-Order Correlation test) (Figure 3). The correlation between miR-671-5p and CHPF2 expression was not significant (*r* = 0.0077, *p* = 0.957, *n* = 51, Spearman's Rank-Order Correlation test) (Figure 3). Levels of CDR1-AS, CDR1 and VSNL1 transcripts significantly decreased or increased in DBTRG, SNB19 and U-87 MG following transfection with miR-671-5p mimics or inhibitors, respectively (Figure 4).

### MiR-671-5p stimulates cell migration and proliferation in GBM DBTRG, SNB-19 and U-87 MG cell lines

**Figure 5:**
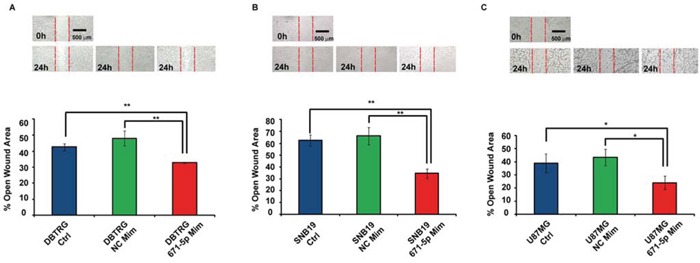
Involvement of miR-671-5p in DBTRG, SNB-19, U-87 MG migration MiR-671-5p mimics stimulate cell migration with respect to scramble molecules (NC Mim) - transfected DBTRG **A.** SNB-19 **B.** U-87 MG **C.** cells in a wound-healing assay. Data are represented as mean percentage ± Standard Deviation of open wound area of three independent biological replicates, 24 h after scratch. Magnification: × 8. Results are representative of at least three randomly selected areas, assayed for each well. *p-value < 0.05; **p-value < 0.01, Student's *t*-test.

**Figure 6:**
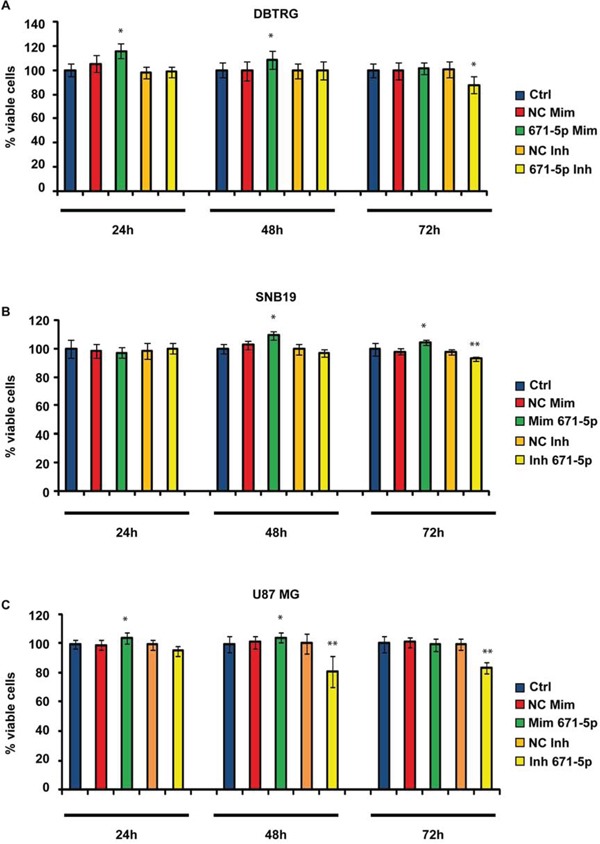
MTT assay in GBM cell lines DBTRG **(A),** SNB-19 **(B),** U-87 MG **(C)**. Data are reported as percentages of viable cells, relative to controls. Experiments were performed as six biological replicates. *P < 0.05, **P < 0.01, Student's *t*-test. Ctrl (not transfected cells); NC Mim (Cells transfected with scramble molecules of miRNA mimics); 671–5p Mim (Cells transfected with miR-671-5p mimics); NC Inh (Cells transfected with scramble molecules of miRNA inhibitors); 671–5p Inh (Cells transfected with miR-671-5p inhibitors).

DBTRG, SNB-19 and U-87 MG transfected with miR-671-5p mimics significantly increased their migration rate of 15%, 32% and 15%, respectively, 24 h after scratching, as compared to matched scramble-transfected cells (Figure 5). DBTRG cells transfected with miR-671-5p mimics significantly increased their viability (16%) at 24 h after transfection; on the other hand, the same cells treated with miR-671-5p inhibitors significantly decreased their viability (12%) 72 h after transfection. SNB-19 showed a significant increase of viability (9%) 48 h after transfection with miR-671-5p mimics and a significant reduction of viability (7%) 72 h after transfection with miR-671-5p inhibitors. U-87 MG significantly increased their viability (5%) 48 h after transfection with miR-671-5p mimics, while significantly decreased viability (19%) 48 h after transfection with miR-671-5p inhibitors (Figure 6).

### Network generation and analysis

**Figure 7:**
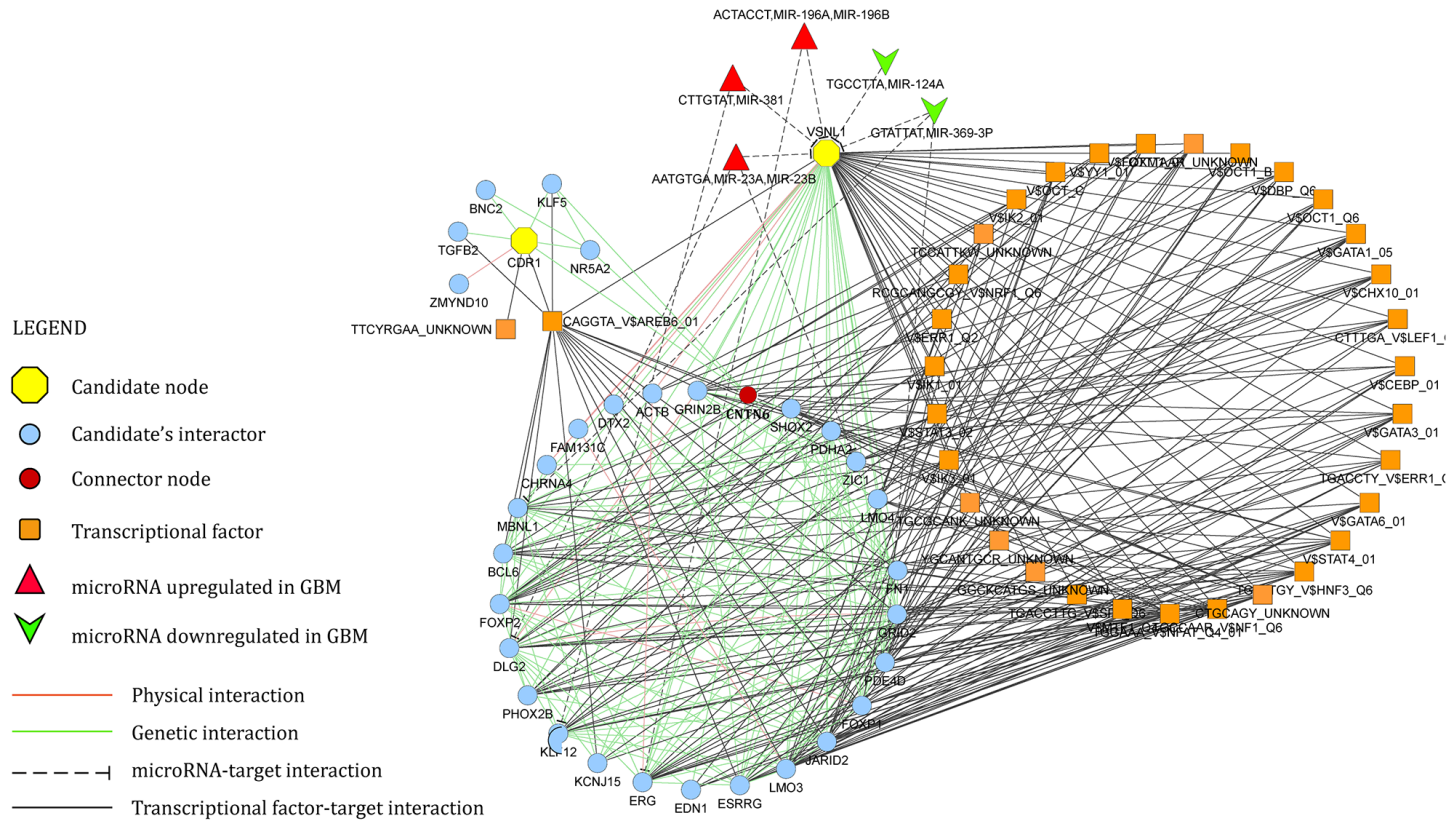
Network of CDR1 and VSNL1 (candidate nodes) plus their first neighbor interactants See legend within the figure and text for further details.

Starting from CDR1 and VSNL1 as input nodes, GeneMANIA algorithm generated a network of 240 nodes and 3935 edges. Edges included validated physical (protein-protein interaction) and genetic interactions, in addition to predicted microRNA-target and transcriptional factor-target interactions. CentiScaPe plug-in v.2.1 applied to the whole network revealed 16 nodes, whose centrality values were equal or higher than thresholds for all selected parameters: Betweenness, Bridging, Closeness, Degree. VSNL1 turned out to be a hub node. Literature mining showed that 15 among the most central nodes in the network are causally involved in the pathogenesis of GBM (specifically, in survival, proliferation, migration and invasion of GBM cells) (Table 1). Functional enrichment analysis performed by ClueGO v2.1.5 on the whole network revealed a statistically significant enrichment in terms like glioma KEGG pathway, central nervous system development, gliogenesis, negative regulation of neuronal apoptosis (see Supplementary Figure 1). The union of the subnetworks of CDR1 and VSNL1 plus their first neighbor interactants generated a new network consisting of 71 nodes and 357 edges (Figure 7). Within this more restricted network, we pinpointed contactin 6 (CNTN6) as the only node that links CDR1 and VSNL1 by a genetic interaction, in addition to 7 miRNAs (miR-23a, miR-23b, miR-124a, miR-196a, miR-196b, miR-369–3p, miR-381) controlling different nodes.

**Table 1:** *Hub nodes* within miR-671-5p targets’ network

Node name	Betweenness	Bridging	Closeness	Degree	GBM reference
BNC2	349.06	4.89	0.0023	48	PMID: 24607568
CAGGTA_V$AREB6_01 [ZEB1]	4973.51	5.29	0.0029	135	PMID: 23818228
CTTTGA_V$LEF1_Q2 [LEF1]	1576.5	4.76	0.0025	83	PMID: 25128061
CXCR4	249.93	5.46	0.0022	36	PMID: 12388552
ETV1	250.47	5.3	0.0022	39	PMID: 19148472
GAB2	296.42	4.81	0.0023	46	PMID: 23231021
GRID2	836.94	4.94	0.0025	73	PMID: 19011622
JAG1	583.17	4.96	0.0024	64	PMID: 22296176
LMO3	598.75	4.98	0.0024	62	PMID: 25829251
NR1D1	269.82	4.84	0.0023	45	PMID: 19011622
SRPK2	293.81	5.59	0.0022	36	PMID: 19011622
TGGAAA_V$NFAT_Q4_01 [NFATC1, NFATC2]	3691.69	4.73	0.0028	129	PMID: 23762456
V$FOXM1_01 [FOXM1]	456.43	4.98	0.0022	46	PMID: 22977194
V$GATA1_05 [GATA1]	1031.95	4.76	0.0024	65	http://digitalcommons.wayne.edu/oa_dissertations/87
V$GATA3_01 [GATA3]	555.79	5.02	0.0022	50	N/A
VSNL1	1411.43	8.66	0.0024	63	PMID: 20525252
**Threshold**	**221.74**	**4.71**	**0.0021**	**32.79**	

## DISCUSSION

MiR-671-5p marked overexpression in GBM and its downstream effects on GBM cells migration and viability strongly suggest that this miRNA is a new oncomiR in GBM. There also are other evidences in literature that support this proposal [14–16]. Based on our *in silico* and experimental data, we further propose that CDR1-AS and VSNL1 are direct miR-671-5p targets, downexpressed in GBM (see Supplementary Figure 2). We also propose that CDR1 is an indirect target of miR-671-5p in the same biological context: its downregulation could be consequent to miR-671-5p-mediated degradation of CDR1-AS, as also suggested by literature [12]. CDR1-AS belongs to the recently discovered class of circRNAs: among them, it is the only one known to be degraded by a miRNA: miR-671-5p [12]. CircRNAs expression appears to be frequently altered in cancer [17]. CDR1-AS has been proposed as candidate gene in Alzheimer's Disease [18] and Diabetes Mellitus [19], but no data have been published on its potential involvement in GBM. Its downregulation, mediated by increased expression of miR-671-5p and associated to downregulation of CDR1, paves the way to the study of new pathways involved in GBM pathogenesis. CDR1 downregulation (likely linked to miR-671-5p-dependent CDR1-AS degradation) may indeed contribute to loss of differentiation of neural cells, leading to neoplastic transformation [20]. On the other hand, VSNL1 is known to inhibit cell migration and behaves as tumour suppressor in several cancer types [21–23]: its downregulation may lead to increased migration rate in GBM cells. Integration of CDR1 and VSNL1 within a physical and genetic interaction network strongly supports our hypothesis on their involvement in GBM: the network is centered on 16 nodes (*hub nodes*), 15 of which are involved in glioma cell pathways and in biological functions as central nervous system development, gliogenesis, negative regulation of neuron apoptosis (see Table 1 and Supplementary Figure 1). VSNL1, a hub node within the network, is predicted to be targeted by 7 miRNAs, 5 of which are oncomiRNAs highly expressed in GBM (miR-23a, miR-23b, miR-196a, miR-196b, miR-381) (Figure 7) [24–28]. This circuitry may be upstream fostered by SOX2 (Sry-related box-2) in GBM cells: this transcription factor is known to promote malignancy in GBM and to positively control the expression of miR-671-5p [29, 30]. Interestingly, literature mining and target analysis reveal that both miR-671-5p and SOX-2 may contribute to inhibiting the expression of common targets, like Solute Carrier Family 7 (Cationic Amino Acid Transporter, y+ System) member 1 (SLC7A1) or Fibronectin type III domain-containing 5 (FNDC5) [29, 30]. FNDC5 was earlier described as involved in neural cell differentiation [31].

## MATERIALS AND METHODS

### Cell cultures and transfection

A172, CAS-1, DBTRG, HCT-116, SK-N-BE, SNB-19, U-87 MG cell lines were obtained from the Interlab Cell Line Collection (ICLC), an International Repository Authority within IST (IRCCS Azienda Ospedaliera Universitaria San Martino- IST - Istituto Nazionale per la Ricerca sul Cancro, Genova, Italy, EU). A375 cell line was purchased from American Type Culture Collection (ATCC). HCT-116 and SK-N-BE cells were grown as previously reported [32, 33]. Further details on culture conditions of other cell lines are provided in Supplementary Methods. Characterization and validation of cell lines were performed by the cell banks. Cell lines were verified to be mycoplasma-free by Hoechst staining and PCR (TIB Molbiol) and by MycoTect (Gibco BRL). Species verification was performed by isoenzyme analysis (AuthentiKit TM System, Innovative Chemistry). Further, profiling of multiplex Short Tandem Repeats was performed to verify identity and uniqueness of cell lines. After receiving the cells, an aliquot was immediately frozen, whereas another was cultured up to the 10^th^ passage to perform the experiments. MiR-671-5p mimics and inhibitors (anti-miR-671-5p) were purchased from Lifetechnologies™ (Carlsbad, CA, USA). For transfection experiments, cells were seeded at a density of 50000 per well in a 24-well plate and transfected using siPORT™ NeoFX™ (LifeTechnologies™). Final concentrations of miR-671-5p mimics and inhibitors were determined according to the best knock-in or knock-down efficiency, for each transfected cell line (see Supplementary Figure 3).

### Cohort of GBM patients

Forty-five paraffin-embedded biopsy samples from GBM patients (GBM: WHO Grade IV) were obtained by the Pathology Laboratory of the Department of Advanced Technologies in Medical and Surgical Sciences *G.F. Ingrassia*, University of Catania, Catania, Italy, EU. The study was approved by the local ethical committee. Cases were selected on the basis of availability of adequate tumor tissue: haematoxylin- and eosin- (H&E) stained slides were reviewed and a concordant diagnosis was performed by trained pathologists, based on WHO 2007 tumor classification [1]. To avoid contamination with non-tumor tissue, the tumor areas were macrodissected with sterile disposable scalpels and then subjected to RNA isolation. Before extraction of total RNA from tumor tissue, each macrodissected section was stained with haematoxylin and eosin for the evaluation of purity of the biopsy. Employing this procedure, only tumor tissue was included and evaluated in the study. Demographic data, clinical and pathological parameters on GBM patients are reported in Supplementary Table 3. Three paraffin-embedded control brain biopsy samples were obtained from the frontal cerebral area as non-neoplastic reference tissues. We avoided using control tissues adjacent to the tumor, since there is the possibility of undetected tumor cell infiltration. Data on EGFR amplification and protein expression were obtained by Fluorescence *In Situ* Hybridization (FISH) and immunohistochemistry (IHC) (see Supplementary Table 3) [34]. Data on O-6-methylguanine-DNA methyltransferase (MGMT) methylation were obtained as previously reported (see Supplementary Table 3) [35]. Volumetric data on biopsies were retrieved through volumetric analysis of post-operative gadolinium (Gd)-enhanced magnetic resonance (MR) images, obtained within 48 h following surgery (see Supplementary Table 3) [35].

### RNA isolation and quantitative real-time RT-PCR

Total RNA from formalin-fixed paraffin-embedded (FFPE) biopsies and from cell lines was purified using RecoverAll™ Total Nucleic Acid Isolation Kit (LifeTechnologies™) and TRIzol^®^ (LifeTechnologies™), respectively. Total whole Brain and Astrocytes RNA was from FirstChoice^®^ Human Total RNA Survey Panel (Ambion^®^, Austin, TX) and ScienCell Research Laboratories^®^ (San Diego, CA), respectively. RNA was quantified and treated with DNase I Amplification Grade (LifeTechnologies™). 30ng of DNase-treated RNA were reverse transcribed by using specific RT miRNA primers and amplified with specific TaqMan^®^ MicroRNA assays (LifeTechnologies™). MiR-92a and miR-99a resulted the most stable miRNAs by applying GeNORM [36]: accordingly, they were used as reference miRNA genes (see Figure 1 legend). 50 ng of DNase-treated RNA from GBM biopsies were reverse transcribed and amplified with target-specific primers in a one-step quantitative real-time PCR reaction through *Power* SYBR^®^ Green RNA-to-C_T_™ *1-Step* Kit (LifeTechnologies™). 1 μg of DNase-treated RNA from cell lines was reverse transcribed through High-Capacity RNA-to-cDNA™ Kit (LifeTechnologies™) and 20 ng of the resulting cDNAs were amplified with target-specific primers by SYBR^®^ Green Master Mix (LifeTechnologies™). TBP was used as reference gene, as it resulted the most stable gene on applying GeNORM. Primer sequences are available upon request. Relative quantification of gene expression was calculated using the comparative Ct (2^−ΔΔCt^) method [37]. We considered differential all the expression values that were statistically significant different between control and test conditions, as previously described [38, 39].

### Western blot analysis

Protein lysates from GBM cell lines were obtained as previously described [39]. Human brain cerebral cortex protein medley was purchased from Takara Clontech ^®^ (Mountain View, CA, USA). 40 μg of total protein extract were loaded into Bolt™ 4–12% Bis-Tris Plus Gel (Lifetechnologies™) and blotted to nitrocellulose membranes by iBlot Dry Blotting System (Lifetechnologies™). Membranes were probed with polyclonal antibodies to CHPF2 (Sigma-Aldrich^®^, Saint Louis, MO, USA) [tested at http://www.proteinatlas.org/ENSG00000033100-CHPF2/antibody] and monoclonal antibodies to VSNL1 (Abcam^®^, Cambridge, UK, EU) and β-actin (Sigma-Aldrich^®^) [38]. β-actin was used as loading control. Proteins were detected by using ECL™ Western Blotting Detection Reagents (GE Healthcare^©^).

### miR-671-5p targets selection

Validated targets of miR-671-5p were retrieved from miRTarbase, release 4.5 [40]. Top 200 predicted targets of miR-671-5p were obtained through TargetScanHuman online algorithm, release 6.2 [41]. The list of predicted targets was further filtered by removing genes: (i) whose expression did not negatively correlate with that of miR-671-5p; (ii) had a miRanda-mirSVR score > - 0.5. Negative correlation among putative targets and miR-671-5p was analyzed through miRGator v.3.0 (http://mirgator.kobic.re.kr) [42]. MiRanda-mirSVR scores (available at http://www.microrna.org) indicate the probability that a miRNA *vs* predicted target alignment lead to downregulation of the latter: more negative is the score, more stringent is the prediction [43]. Final selection of putative targets for downstream experimental analysis has been based on literature mining, following these criteria: (i) unknown involvement in GBM pathogenesis; (ii) tumor suppressor genes with known correlation with cancer, neuronal differentiation or cell migration. Tumor suppressor genes were retrieved within the list of predicted targets by Tumor Suppressor Gene Database (http://bioinfo.mc.vanderbilt.edu/TSGene/).

### Wound-healing assay

Cells were plated in 24-well plates and grown to 80%-90% confluence by using the same transfection protocol described above. A wound was created in the cell layer by using a pipette tip. Cells were washed twice with PBS to remove cell debris and floating cells and were refeeded with new serum-free medium for 24 h. Wounds were subsequently observed under an inverted microscope (Leitz, Fluorvert FU): images covering the entire width of the wounds were captured on camera (Leica, DFC 495), at time zero and after 24 h, by using an 8 × objective. TScratch v. 1.0 software was used to calculate the percentage of open wound area for each condition. At least three randomly selected areas were assayed for each well. Results are shown as the mean ± standard deviation of the percentage of open wound area of three biological replicates.

### MTT assay

Cell viability was evaluated through MTT assay. Briefly, 10^4^ cells / well were reverse transfected with miR-671-5p mimics or inhibitors or scramble molecules, seeded into 96-well plates and incubated at 37°C for 24, 48, 72 h. At the end of each time point, cells were incubated for 3 h with 5 mg/ml of MTT solution (serum-free medium was used as solvent). Washing with phosphate-buffered saline (PBS) was followed by addition of dimethyl sulfoxide (DMSO). The solution was gently shaken for 10′, so that complete dissolution of formazan crystals was achieved. Absorbance was recorded at 550 nm, using the microplate spectrophotometer system Multiscan Ascent^®^ microplate reader (Thermo Fisher Scientific). Data were exported and analyzed through Excel. Final results are presented as the mean ± standard deviation of the percentage of cell viability of six biological replicates.

### Network generation and analysis

A network of physical and genetic interactions was generated by GeneMANIA plug-in [44, 45] in Cytoscape v3.2.0 [46]. Specifically, CDR1 and VSNL1 were given as input to GeneMANIA plug-in. Network centrality parameters Betweenness, Bridging centrality, Closeness and Degree were analyzed through CentiScaPe plug-in v.2.1 [47]. All the nodes having network centrality parameter values above the mean (threshold) were considered *hubs* of the network. Functional enrichment analysis (Gene ontologies and KEGG pathways) among the nodes of the network was performed through the Cytoscape plug-in ClueGO v2.1.5 [48].

### Statistical analysis

Comparison between two experimental groups was performed through Student's *t*-test. To compare three or more groups, we used one way ANOVA. Spearman Rank-Order Correlation Coefficient was calculated to correlate gene expression values. *P*-values < 0.05 were considered to be statistically significant.

## SUPPLEMENTARY METHODS FIGURES AND TABLES








