# Supplementary material for: Dysregulated miR-671-5p / CDR1-AS / CDR1 / VSNL1 axis is involved in glioblastoma multiforme
Source: Oncotarget. 2015 Dec 15;7(4):4746–59. doi: 10.18632/oncotarget.6621 (PMC4826240; doi:10.18632/oncotarget.6621)
Supplement: Supplementary file 1 [file oncotarget-07-4746-s001.pdf]

SUPPLEMENTARY METHODS

Cell cultures

A172 human glioblastoma cells were grown in DMEM with 4.5 g/L glucose supplemented with 10% FBS, 2 mM L-Glutamine, 1 mM sodium pyruvate; CAS-1 and SNB-19 cells were grown in DMEM with 1 g/L glucose supplemented with 10% FBS, 2 mM L-Glutamine; DBTRG cells were grown in RPMI 1640 with L-Glutamine, supplemented with 10% FBS; U-87 MG cells were grown in DMEM with 1 g/L glucose supplemented with 10% FBS, 1% non-essential amino acids 1 mM sodium pyruvate; A375 melanoma cells were grown in DMEM with 4.5 g/L glucose supplemented with 10% FBS, 2 mM L-Glutamine.

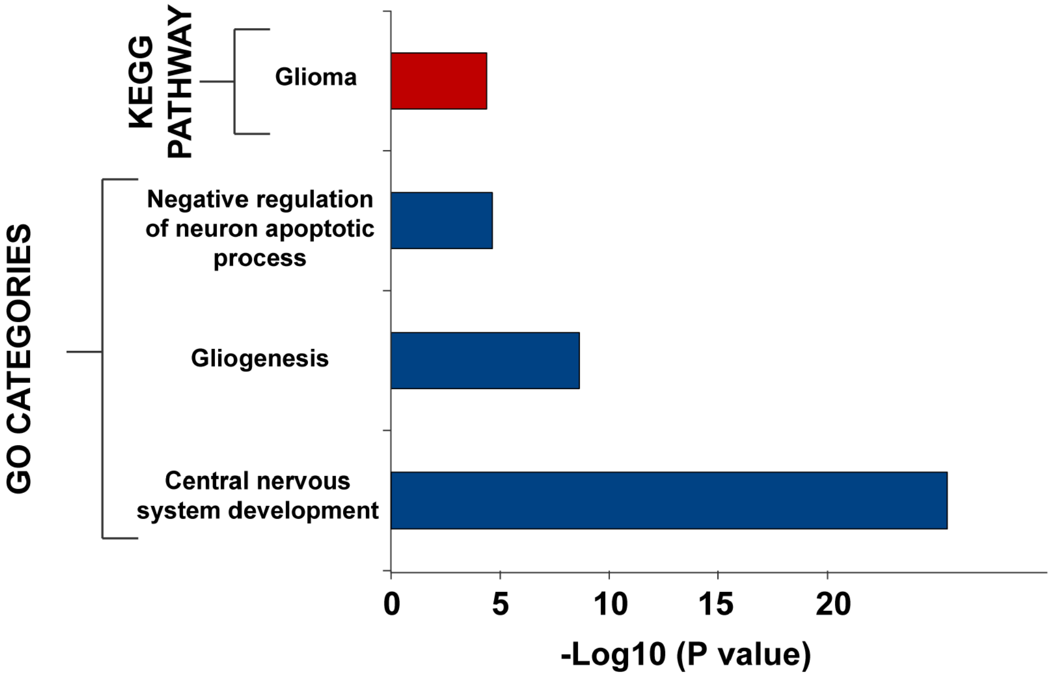

Supplementary Figure S1: Enriched pathways and biological processes within miR-671-5p targets' network. *P*-value were calculated with Fisher Exact Test and corrected through Benjamini-Hochberg method.

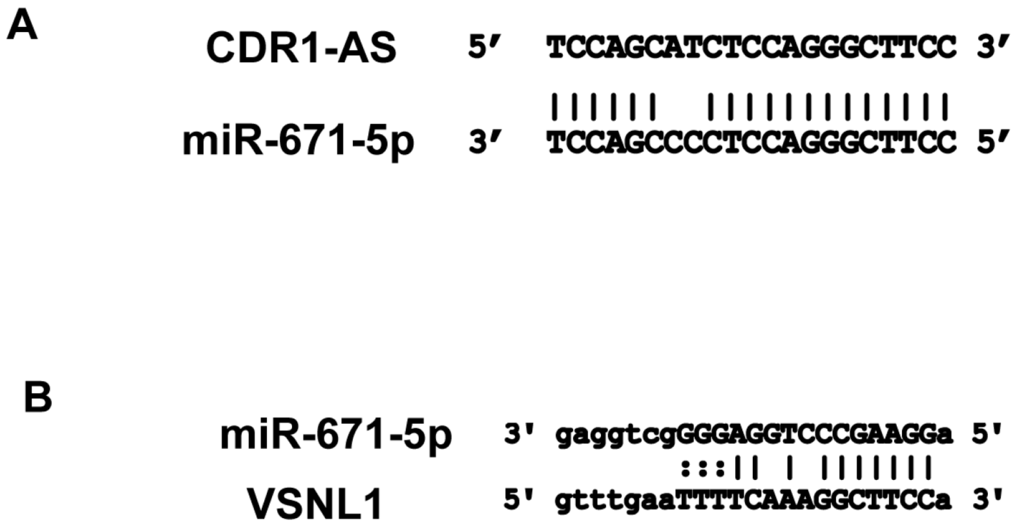

Supplementary Figure S2: MiR-671-5p / target alignments. A. Alignment between miR-671-5p and CDR1-AS RNA sequences (performed through Nucleotide BLAST, http://blast.ncbi.nlm.nih.gov/). **B.** Alignment between miR-671-5p and VSNL1 RNA sequences as appeared by http://microrna.org (http://www.microrna.org/) output.

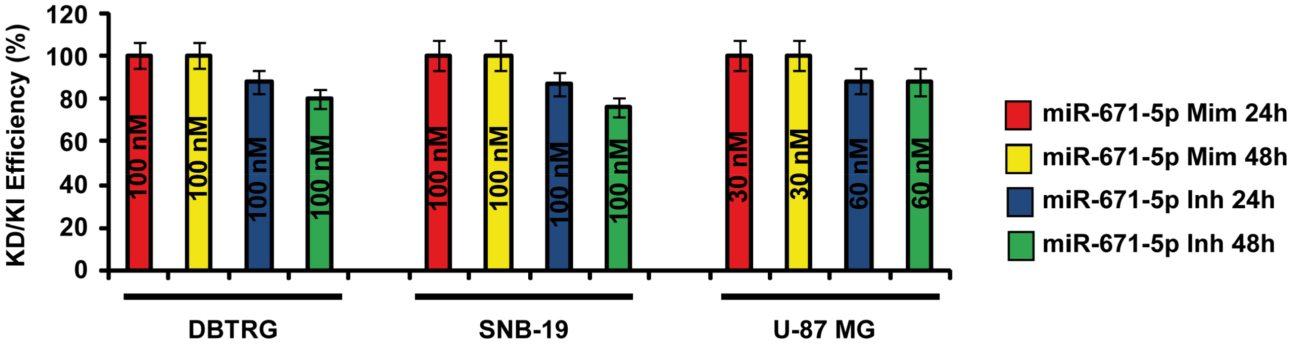

Supplementary Figure S3: MiR-671-5p knock-down (KD) and knock-in (KI) efficiencies (%) after transfection of DBTRG, SNB-19 and U-87 MG with miRNA mimics (Mim) or inhibitors (Inh). Data are reported as mean ± standard deviation of three independent experiments.

Supplementary Table S1: Validated targets of *miR-671-5p*

Supplementary Table S2: Predicted targets of *miR-671-5p*

Supplementary Table S3: GBM patients' clinical data
