# Supplementary material for: Dysregulated miR-671-5p / CDR1-AS / CDR1 / VSNL1 axis is involved in glioblastoma multiforme
Source: Oncotarget. 2015 Dec 15;7(4):4746–59. doi: 10.18632/oncotarget.6621 (PMC4826240; doi:10.18632/oncotarget.6621)
Supplement: Supplementary file 2 [file oncotarget-07-4746-s002.doc]

| **Table S1 Validated targets of *miR-671-5p*** | | |  |  |  |
| --- | --- | --- | --- | --- | --- |
|  |  |  |  |  |  |
| # | **Target Gene** | **Full name** | **Experiments** | **Experimental model** | **References (PMID)** |
| 1 | CDR1-AS | cerebellar degeneration-related protein 1(CDR1) antisense RNA | Luciferase reporter assay/Microarray/Northern blot/qRT-PCR | HEK293 Cell Line | 21964070 |
| 2 | GPI | glucose phosphate isomerase | CLASH | Flp-In T-REx-293 Cell Line | 23622248 |
| 3 | CRLF3 | cytokine receptor-like factor 3 | CLASH | Flp-In T-REx-293 Cell Line | 23622248 |
| 4 | RPL23A | ribosomal protein L23a | CLASH | Flp-In T-REx-293 Cell Line | 23622248 |
| 5 | CEP85 | centrosomal protein 85kDa | CLASH | Flp-In T-REx-293 Cell Line | 23622248 |
| 6 | NACA | nascent polypeptide-associated complex alpha subunit | CLASH | Flp-In T-REx-293 Cell Line | 23622248 |
| 7 | SSPO | SCO-spondin | CLASH | Flp-In T-REx-293 Cell Line | 23622248 |
| 8 | UBE2C | ubiquitin-conjugating enzyme E2C | CLASH | Flp-In T-REx-293 Cell Line | 23622248 |
| 9 | RREB1 | ras responsive element binding protein 1 | CLASH | Flp-In T-REx-293 Cell Line | 23622248 |
| 10 | C17orf58 | chromosome 17 open reading frame 58 | CLASH | Flp-In T-REx-293 Cell Line | 23622248 |
| 11 | NRSN2 | neurensin 2 | CLASH | Flp-In T-REx-293 Cell Line | 23622248 |
| 12 | CAPN15 (Alias SOLH) | calpain 15 | CLASH | Flp-In T-REx-293 Cell Line | 23622248 |
| 13 | COA4 | cytochrome c oxidase assembly factor 4 homolog | CLASH | Flp-In T-REx-293 Cell Line | 23622248 |
| 14 | HIST2H2AA3 | histone cluster 2, H2aa3 | CLASH | Flp-In T-REx-293 Cell Line | 23622248 |
| 15 | SLC7A5 | solute carrier family 7 (cationic amino acid transporter, y+ system), member 5 | CLASH | Flp-In T-REx-293 Cell Line | 23622248 |
| 16 | CDC123 | cell division cycle 123 | CLASH | Flp-In T-REx-293 Cell Line | 23622248 |
| 17 | TXNRD2 | thioredoxin reductase 2 | CLASH | Flp-In T-REx-293 Cell Line | 23622248 |
| 18 | ZFP36L2 | zinc finger protein 36, C3H type-like 2 | CLASH | Flp-In T-REx-293 Cell Line | 23622248 |
| 19 | FBRS | fibrosin | CLASH | Flp-In T-REx-293 Cell Line | 23622248 |
| 20 | OGT | O-linked N-acetylglucosamine (GlcNAc) transferase | PAR-CLIP/CLASH | HEK293 Cell Line and derivatives | 23622248/20371350/21572407/23446348 |
| 21 | NDUFA3 | NADH dehydrogenase (ubiquinone) 1 alpha subcomplex, 3, 9kDa | CLASH | Flp-In T-REx-293 Cell Line | 23622248 |
| 22 | HMGA1 | high mobility group AT-hook 1 | CLASH | Flp-In T-REx-293 Cell Line | 23622248 |
| 23 | OXA1L | oxidase (cytochrome c) assembly 1-like | CLASH | Flp-In T-REx-293 Cell Line | 23622248 |
| 24 | AGO2 | argonaute RISC catalytic component 2 | CLASH | Flp-In T-REx-293 Cell Line | 23622248 |
| 25 | SLC7A1 | solute carrier family 7 (cationic amino acid transporter, y+ system), member 1 | PAR-CLIP/CLASH | Flp-In T-REx-293 Cell Line | 20371350/23622248 |
| 26 | ATP2A2 | ATPase, Ca++ transporting, cardiac muscle, slow twitch 2 | CLASH | Flp-In T-REx-293 Cell Line | 23622248 |
| 27 | XRCC6 | X-ray repair complementing defective repair in Chinese hamster cells 6 | CLASH | Flp-In T-REx-293 Cell Line | 23622248 |
| 28 | RPL3 | ribosomal protein L3 | CLASH | Flp-In T-REx-293 Cell Line | 23622248 |
| 29 | LCT | lactase | CLASH | Flp-In T-REx-293 Cell Line | 23622248 |
| 30 | BAZ2A | bromodomain adjacent to zinc finger domain, 2A | PAR-CLIP/CLASH | HEK293 Cell Line and derivatives | 21572407/23622248 |
| 31 | HDAC1 | histone deacetylase 1 | CLASH | Flp-In T-REx-293 Cell Line | 23622248 |
| 32 | PRR14L | proline rich 14-like | CLASH | Flp-In T-REx-293 Cell Line | 23622248 |
| 33 | METTL23 | methyltransferase like 23 | CLASH | Flp-In T-REx-293 Cell Line | 23622248 |
| 34 | CHPF2 | chondroitin sulfate glucuronyltransferase | CLASH | Flp-In T-REx-293 Cell Line | 23622248 |
| 35 | R3HDM2 | R3H domain containing 2 | CLASH/HITS-CLIP | Flp-In T-REx-293 Cell Line/293S | 23622248/23824327 |
| 36 | LGALS3BP | lectin, galactoside-binding, soluble, 3 binding protein | PAR-CLIP/CLASH | TZM-BL/HEK293 Cell Line | 23592263/23622248 |
| 37 | USP21 | ubiquitin specific peptidase 21 | CLASH | Flp-In T-REx-293 Cell Line | 23622248 |
| 38 | MEPCE | methylphosphate capping enzyme | CLASH | Flp-In T-REx-293 Cell Line | 23622248 |
| 39 | POR | P450 (cytochrome) oxidoreductase | CLASH | Flp-In T-REx-293 Cell Line | 23622248 |
| 40 | LRRN2 | leucine rich repeat neuronal 2 | CLASH | Flp-In T-REx-293 Cell Line | 23622248 |
| 41 | INPPL1 | inositol polyphosphate phosphatase-like 1 | CLASH | Flp-In T-REx-293 Cell Line | 23622248 |
| 42 | CRTC3 | CREB regulated transcription coactivator 3 | HITS-CLIP/CLASH | HeLa/Flp-In T-REx-293 Cell Line | 23313552/23622248 |
| 43 | MOSPD3 | motile sperm domain containing 3 | CLASH | Flp-In T-REx-293 Cell Line | 23622248 |
| 44 | HNRNPUL1 | heterogeneous nuclear ribonucleoprotein U-like 1 | CLASH | Flp-In T-REx-293 Cell Line | 23622248 |
| 45 | U2AF2 | U2 small nuclear RNA auxiliary factor 2 | CLASH | Flp-In T-REx-293 Cell Line | 23622248 |
| 46 | HJURP | Holliday junction recognition protein | CLASH | Flp-In T-REx-293 Cell Line | 23622248 |
