# Supplementary material for: Dysregulated miR-671-5p / CDR1-AS / CDR1 / VSNL1 axis is involved in glioblastoma multiforme
Source: Oncotarget. 2015 Dec 15;7(4):4746–59. doi: 10.18632/oncotarget.6621 (PMC4826240; doi:10.18632/oncotarget.6621)
Supplement: Supplementary file 3 [file oncotarget-07-4746-s003.doc]

| **Table S2 Predicted targets of *miR-671-5p*** | | |  |  |  |  |
| --- | --- | --- | --- | --- | --- | --- |
|  |  |  |  |  |  |  |
| **#** | **Predicted Target** | **Official Gene Symbol** | **Full name** | **TargetScan Conserved sites (total)** | **TargetScan Poorly conserved sites (total)** | **mirSVR SCORE** |
| 1 | SLC30A6 | SLC30A6 | solute carrier family 30 (zinc transporter), member 6 | 0 | 3 | -3.6715 |
| 2 | ST8SIA5 | ST8SIA5 | ST8 alpha-N-acetyl-neuraminide alpha-2,8-sialyltransferase 5 | 0 | 2 | -2.1409 |
| 3 | CFL2 | CFL2 | cofilin 2 (muscle) | 0 | 3 | -1.9219 |
| 4 | KRT9 | KRT9 | keratin 9, type I | 0 | 2 | -1.8948 |
| 5 | SATB2 | SATB2 | SATB homeobox 2 | 0 | 3 | -1.7189 |
| 6 | C12orf77 | C12orf77 | hypothetical protein LOC196415 | 0 | 2 | -1.4802 |
| 7 | C16orf72 | C16orf72 | chromosome 16 open reading frame 72 | 2 | 0 | -1.4362 |
| 8 | C12orf69 | SMCO3 | single-pass membrane protein with coiled-coil domains 3 | 0 | 1 | -1.3242 |
| 9 | ACBD6 | ACBD6 | acyl-Coenzyme A binding domain containing 6 | 0 | 1 | -1.319 |
| 10 | TBL2 | TBL2 | transducin (beta)-like 2 | 0 | 2 | -1.3185 |
| 11 | SSBP1 | SSBP1 | single-stranded DNA binding protein 1, mitochondrial | 0 | 1 | -1.3111 |
| 12 | FTSJ1 | FTSJ1 | FtsJ RNA methyltransferase homolog 1 (E. coli) | 0 | 1 | -1.295 |
| 13 | RBMS3 | RBMS3 | RNA binding motif, single stranded interacting protein 3 | 1 | 1 | -1.2569 |
| 14 | C9orf78 | C9orf78 | chromosome 9 open reading frame 78 | 0 | 2 | -1.2475 |
| 15 | VSNL1 | VSNL1 | visinin-like 1 | 0 | 1 | -1.1897 |
| 16 | FCAMR | FCAMR | Fc receptor, IgA, IgM, high affinity | 1 | 0 | -1.1865 |
| 17 | SGK2 | SGK2 | serum/glucocorticoid regulated kinase 2 | 1 | 1 | -1.184 |
| 18 | RAB32 | RAB32 | RAB32, member RAS oncogene family | 0 | 1 | -1.1149 |
| 19 | DLC1 | DLC1 | DLC1 Rho GTPase activating protein | 0 | 2 | -1.1115 |
| 20 | SORBS2 | SORBS2 | sorbin and SH3 domain containing 2 | 0 | 1 | -1.1022 |
| 21 | FAM177A1 | FAM177A1 | family with sequence similarity 177, member A1 | 0 | 1 | -1.0785 |
| 22 | SLC6A16 | SLC6A16 | solute carrier family 6, member 16 | 0 | 1 | -1.0408 |
| 23 | GOLIM4 | GOLIM4 | golgi integral membrane protein 4 | 0 | 1 | -1.0195 |
| 24 | PTCHD1 | PTCHD1 | patched domain containing 1 | 0 | 2 | -1.0129 |
| 25 | KRT38 | KRT38 | keratin 38, type I | 0 | 2 | -0.9976 |
| 26 | WFDC6 | WFDC6 | WAP four-disulfide core domain 6 | 0 | 1 | -0.9863 |
| 27 | KCTD16 | KCTD16 | potassium channel tetramerisation domain containing 16 | 1 | 0 | -0.9645 |
| 28 | SPINLW1 | EPPIN | epididymal peptidase inhibitor | 1 | 1 | -0.9627 |
| 29 | SPTBN2 | SPTBN2 | spectrin, beta, non-erythrocytic 2 | 0 | 1 | -0.9456 |
| 30 | USP36 | USP36 | ubiquitin specific peptidase 36 | 0 | 1 | -0.9397 |
| 31 | SASH3 | SASH3 | SAM and SH3 domain containing 3 | 1 | 1 | -0.9264 |
| 32 | FBXL22 | FBXL22 | F-box and leucine-rich repeat protein 22 | 0 | 1 | -0.9099 |
| 33 | TRIM47 | TRIM47 | tripartite motif containing 47 | 0 | 1 | -0.8819 |
| 34 | KIT | KIT | v-kit Hardy-Zuckerman 4 feline sarcoma viral oncogene homolog | 0 | 2 | -0.8744 |
| 35 | ANKS1A | ANKS1A | ankyrin repeat and sterile alpha motif domain containing 1A | 1 | 0 | -0.869 |
| 36 | KLHL21 | KLHL21 | kelch-like family member 21 | 0 | 2 | -0.8485 |
| 37 | DDX21 | DDX21 | DEAD (Asp-Glu-Ala-Asp) box helicase 21 | 0 | 2 | -0.841 |
| 38 | ATP5SL | ATP5SL | ATP5S-like | 0 | 2 | -0.82 |
| 39 | NDUFB5 | NDUFB5 | NADH dehydrogenase (ubiquinone) 1 beta subcomplex, 5, 16kDa | 0 | 1 | -0.8156 |
| 40 | ANKRD46 | ANKRD46 | ankyrin repeat domain 46 | 0 | 1 | -0.7459 |
| 41 | HIVEP3 | HIVEP3 | human immunodeficiency virus type I enhancer binding protein 3 | 0 | 1 | -0.7439 |
| 42 | SARS2 | SARS2 | seryl-tRNA synthetase 2, mitochondrial | 0 | 1 | -0.7356 |
| 43 | WASH1 | WASH1 | WAS protein family homolog 1 | 0 | 1 | -0.7159 |
| 44 | EPB41L4B | EPB41L4B | erythrocyte membrane protein band 4.1 like 4B | 0 | 2 | -0.703 |
| 45 | PIP4K2C | PIP4K2C | phosphatidylinositol-5-phosphate 4-kinase, type II, gamma | 0 | 1 | -0.693 |
| 46 | LEP | LEP | leptin | 1 | 0 | -0.6863 |
| 47 | HPCA | HPCA | hippocalcin | 0 | 3 | -0.6672 |
| 48 | FNDC5 | FNDC5 | fibronectin type III domain containing 5 | 2 | 0 | -0.6438 |
| 49 | ZNF627 | ZNF627 | zinc finger protein 627 | 0 | 2 | -0.6427 |
| 50 | BMP8B | BMP8B | bone morphogenetic protein 8b | 0 | 2 | -0.6364 |
| 51 | SYPL2 | SYPL2 | synaptophysin-like 2 | 1 | 2 | -0.6245 |
| 52 | CDR2L | CDR2L | cerebellar degeneration-related protein 2-like | 1 | 2 | -0.6027 |
| 53 | MS4A8B | MS4A8 | membrane-spanning 4-domains, subfamily A, member 8 | 0 | 1 | -0.5905 |
| 54 | PHF17 | JADE1 | jade family PHD finger 1 | 2 | 0 | -0.5799 |
| 55 | FAM45A | FAM45A | family with sequence similarity 45, member A | 0 | 1 | -0.5749 |
| 56 | PDLIM5 | PDLIM5 | PDZ and LIM domain 5 | 0 | 1 | -0.5394 |
| 57 | CLCN5 | CLCN5 | chloride channel 5, voltage-sensitive 5 | 0 | 2 | -0.5273 |
| 58 | RNF38 | RNF38 | ring finger protein 38 | 1 | 0 | -0.5079 |
| 59 | EP400 | EP400 | E1A binding protein p400 | 0 | 2 | -0.5055 |
| 60 | ADAMTSL5 | ADAMTSL5 | ADAMTS-like 5 | 0 | 1 | -0.5044 |
| 61 | LMNA | LMNA | lamin A/C | 0 | 3 | -0.501 |
|  |  |  |  |  |  |  |
| Only predicted targets with mirSVR score ≤-0.5 are shown | | |  |  |  |  |
| Targets are ordered by increasing mirSVR score | | |  |  |  |  |
