# Supplementary material for: Dysregulated miR-671-5p / CDR1-AS / CDR1 / VSNL1 axis is involved in glioblastoma multiforme
Source: Oncotarget. 2015 Dec 15;7(4):4746–59. doi: 10.18632/oncotarget.6621 (PMC4826240; doi:10.18632/oncotarget.6621)
Supplement: Supplementary file 4 [file oncotarget-07-4746-s004.doc]

| **Table S3 GBM patients’ clinical data** | | |  |  |  |  |  |  |  |
| --- | --- | --- | --- | --- | --- | --- | --- | --- | --- |
|  |  |  |  |  |  |  |  |  |  |
| # | **SAMPLE ID** | **MGMT PROMOTER METHYLATION** | **IHC (EGFR)** | **FISH (EGFR)** | **TUMOR SITE** | **TUMOR VOLUME (mm^3^)** | **OS** | **PATIENT’S AGE** | **SEX** |
| 1 | 04/43272-A | N/A | ++ | + | Right occipito-temporal | N/A | 14 Months | 73 | F |
| 2 | 04/49644-A | N/A | + | NORMAL | Right frontal intra-axial | 34.5 | 10 Months | 72 | M |
| 3 | 06/51813-G | Intermediate methylation | + | NORMAL | Right parietal intra-axial | 55.7 | 10 Months | 59 | M |
| 4 | 04/45187-1A | N/A | ++ | + | Right insular fronto-temporal | 48.3 | 20 Months | 67 | M |
| 5 | 06/54956-2C | N/A | - | NORMAL | Left insular fronto-temporal | 43.5 | 15 Months | 65 | M |
| 6 | 05/47756-2R | High methylation | ++ | + | Right temporal | N/A | 40 Months | 69 | M |
| 7 | 06/50051-2C | Unmethylation | ++ | + | Right frontal intra-axial | N/A | 6 Months | 55 | M |
| 8 | 06/51473-2 | N/A | - | NORMAL | Right intra-axial | 15.2 | 5 Months | 68 | M |
| 9 | 06/53090-D | High methylation | - | NORMAL | Left frontal | 27.5 | 93 Months (Living) | 55 | F |
| 10 | 04/43117-3E | N/A | + | + | Right temporal | N/A | 2 Months | 70 | M |
| 11 | 03/48277-A | N/A | - | NORMAL | Right temporal intra-axial | 19.3 | 9 Months | 79 | M |
| 12 | 04/43528-C | N/A | - | NORMAL | Left frontal prerolandic convolution | N/A | 6 Months | 75 | F |
| 13 | 06/50931-C | N/A | ++ | NORMAL | Left temporo-parietal intra-axial | 38.2 | 11 Months | 80 | F |
| 14 | 06/54770-B | N/A | - | NORMAL | Right nuclear temporo-insular | 33.5 | 18 Months | 61 | F |
| 15 | 04/45264-2A | N/A | - | NORMAL | Left temporal cortical-subcortical | N/A | 14 Months | 65 | M |
| 16 | 06/50283-A | N/A | - | NORMAL | Left frontal | 29.5 | 44 Months | 57 | M |
| 17 | 04/42199-A | Unmethylation | + | NORMAL | Right occipito-parietal | N/A | 8 Months | 80 | M |
| 18 | 04/41805F | N/A | - | NORMAL | Right temporo-parietal | N/A | 12 Months | 79 | M |
| 19 | 04/42084B | N/A | - | NORMAL | Right temporal intra-axial | N/A | 31 Months | 49 | M |
| 20 | 04/43545-C | Unmethylation | N/A | N/A | Right paramedian | N/A | 11 Months | 69 | M |
| 21 | 07/58570-A | Unmethylation | N/A | N/A | Right temporal | 18 | 18 Months | 66 | M |
| 22 | 10/81707-B | Unmethylation | N/A | N/A | Right frontal | 59.7 | 8 Months | 82 | F |
| 23 | 11/88812-A | Unmethylation | N/A | N/A | Right temporopolar | 44.9 | 30 Months (Living) | 68 | F |
| 24 | 11/86962-3E | Unmethylation | N/A | N/A | Left frontal | 44.6 | 9 Months | 48 | F |
| 25 | 12/90772-D | Unmethylation | N/A | N/A | Right frontal cortical-subcortical | 3.52 | 7 Months | 73 | M |
| 26 | 12/93238-2 | Unmethylation | N/A | N/A | Right frontal prerolandic | 13.7 | 2 Months | 71 | F |
| 27 | 05/45601-E | Unmethylation | N/A | N/A | Left insular fronto-temporal | N/A | 22 Months | 64 | F |
| 28 | 06/53090-D | High methylation | N/A | N/A | Left frontal | 27.5 | 93 Months (Living) | 55 | F |
| 29 | 10/77746-B | N/A | N/A | N/A | Left temporal | 56.9 | 22 Months | 50 | M |
| 30 | 09/73099-D | High methylation | N/A | N/A | Right frontal | 56.1 | 20 Months | 66 | M |
| 31 | 06/51129-2A | High methylation | N/A | N/A | Right third middle cingulum | 3.87 | 40 Months | 54 | M |
| 32 | 08/66254-2I | Intermediate methylation | N/A | N/A | Left frontal | 14.4 | 7 Months | 74 | F |
| 33 | 10/78321-A | Intermediate methylation | N/A | N/A | Left temporal parahippocampal | 18.2 | 28 Months | 52 | M |
| 34 | 10/76547-2A | Unmethylation | N/A | N/A | Right frontal | 45.2 | 17 Months | 84 | F |
| 35 | 09/73472-2D | High methylation | N/A | N/A | Left frontal | 38.2 | 27 Months | 75 | F |
| 36 | 04/41410-2 | Intermediate methylation | N/A | N/A | Left parietal paraventricular | N/A | 10 Months | 77 | F |
| 37 | 10/75663-2A | Unmethylation | N/A | N/A | Left temporo-insular | 5.24 | 16 Months | 54 | M |
| 38 | 12/90803-A | Unmethylation | N/A | N/A | Left frontal | 0.8 | 7 Months | 60 | F |
| 39 | 12/94538-A5 | Unmethylation | N/A | N/A | Right temporal | 26 | 14 Months | 70 | M |
| 40 | 11/85499-B | Intermediate methylation | N/A | N/A | Left occipito-parietal | 36.6 | 8 Months | 65 | M |
| 41 | 11/85539-2D | High methylation | N/A | N/A | Left temporo-insular | 33.1 | 34 Months | 63 | F |
| 42 | 12/92904-C | High methylation | N/A | N/A | Right frontal | 69.4 | 24 Months (Living) | 65 | F |
| 43 | 12/94201-3 | Unmethylation | N/A | N/A | Right frontal | 24 | 22 Months (Living) | 55 | F |
| 44 | 10/87361-3A | N/A | N/A | N/A | Right parietal | 34 | 11 Months | 68 | M |
| 45 | 10/79332-2A | Intermediate methylation | N/A | N/A | Right occipito-parietal parasagittal | 16.2 | 44 Months (Living) | 64 | M |
|  |  |  |  |  |  |  |  |  |  |
| FISH (EGFR) = Fluorescence In Situ Hybridization for EGFR gene; IHC (EGFR)= Immunohistochemistry for EGFR protein; MGMT (O-6-methylguanine-DNA methyltransferase); N/A (Not available); OS (Overall Survival) | | | | | | | | | |
